# Comparative approaches for assessing access to alcohol outlets: exploring the utility of a gravity potential approach

**DOI:** 10.1186/s12963-016-0097-x

**Published:** 2016-08-02

**Authors:** Tony H. Grubesic, Ran Wei, Alan T. Murray, William Alex Pridemore

**Affiliations:** 1 Center for Spatial Reasoning & Policy Analytics, College of Public Service & Community Solutions, Arizona State University, 411 N. Central Ave, Suite 600, Phoenix, AZ 85004 USA; 2 Department of Geography, University of California – Santa Barbara, Santa Barbara, CA 93106 USA; 3 Department of Geography, University of Utah, 260 S. Central Campus Dr., Salt Lake City, Utah 84112 USA; 4 School of Criminal Justice, University at Albany, State University of New York, 1400 Washington Ave., Albany, NY 12222 USA

## Abstract

**Background:**

A growing body of research recommends controlling alcohol availability to reduce harm. Various common approaches, however, provide dramatically different pictures of the physical availability of alcohol. This limits our understanding of the distribution of alcohol access, the causes and consequences of this distribution, and how best to reduce harm. The aim of this study is to introduce both a gravity potential measure of access to alcohol outlets, comparing its strengths and weaknesses to other popular approaches, and an empirically-derived taxonomy of neighborhoods based on the type of alcohol access they exhibit.

**Methods:**

We obtained geospatial data on Seattle, including the location of 2402 alcohol outlets, United States Census Bureau estimates on 567 block groups, and a comprehensive street network. We used exploratory spatial data analysis and employed a measure of inter-rater agreement to capture differences in our taxonomy of alcohol availability measures.

**Results:**

Significant statistical and spatial variability exists between measures of alcohol access, and these differences have meaningful practical implications. In particular, standard measures of outlet density (e.g., spatial, per capita, roadway miles) can lead to biased estimates of physical availability that over-emphasize the influence of the control variables. Employing a gravity potential approach provides a more balanced, geographically-sensitive measure of access to alcohol outlets.

**Conclusions:**

Accurately measuring the physical availability of alcohol is critical for understanding the causes and consequences of its distribution and for developing effective evidence-based policy to manage the alcohol outlet licensing process. A gravity potential model provides a superior measure of alcohol access, and the alcohol access-based taxonomy a helpful evidence-based heuristic for scholars and local policymakers.

## Background

Evidence shows communities with greater alcohol availability experience a number of distinct negative consequences, including violence, antisocial behavior, sexually transmitted diseases, reduced productivity at work, and general quality of life [1–14]. Research in public health and social sciences supports the availability theory of alcohol [15, 16], which is based on three core tenets: a) as alcohol availability increases in a community, the mean consumption of alcohol within the local population increases; b) as the mean consumption increases, the number of heavy drinkers also increases; and, c) as heavy drinking within the population increases, so do the associated with adverse health and social outcomes. The primary goal of this paper is to deepen our understanding of an important methodological component of this theory – how to measure the physical availability of alcohol – by introducing a gravity potential measure of access to outlets that we believe provides a better methodological foundation for exploring the theoretical concept of availability. To do this we explore the strengths and weaknesses associated with several popular areal measures of the physical availability of alcohol, including basic container indices (e.g., simple counts, outlets weighted by area, etc.), proximity-based measurements (e.g., distance to nearest outlet), and our more advanced gravity-based approach (e.g., spatial interaction models). A second goal is to introduce an empirically-derived taxonomy of neighborhoods based on the type of alcohol access they exhibit.

Our work provides several important contributions to public health, law enforcement, and public policy. First, alcohol availability’s association with social problems like violence plagues large cities [2, 4, 6, 7], small towns [17], and rural communities [18]. Regardless of the geographic setting, the ability to accurately measure the physical availability of alcohol and its relationship with negative outcomes is critical for crafting more informed outlet licensing strategies, public health interventions, and approaches for mitigating community risk. Second, there is no consensus on which measures perform best for this task. These measures also may be context-dependent, where urban settings require a different approach than in rural locales. Thus, without a deeper understanding of how each measure is structured, it is exceedingly difficult to select the appropriate metric(s) for analysis. Finally, as detailed by Holmes et al. [19], there is a need for innovation in measurement approaches for evaluating the physical availability of alcohol and its associated impacts, particularly for policy practitioners. We respond to the call from Holmes et al. by detailing a more sensitive and theoretically informed spatial interaction model that can be used for estimating regional access to alcohol outlets, and we highlight its relative advantages when compared to traditional container and proximity-based measurements. Our approach is not limited to alcohol outlets but can be used to measure the distribution and effects of a wide range of other nuisance facilities like payday lenders [20], pawn shops [21], public transportation nodes [22], abandoned structures [23], and certain types of land use [24].

### Physical availability of alcohol

Although availability theory generally is critical to understanding the potential impacts of alcohol on a populace, measuring access is equally important. In fact, access is an essential component in measuring the general physical availability of alcohol [6, 10, 15, 19]. Stockwell and Gruenewald [15] suggest alcohol availability takes two forms. Economic availability focuses on alcohol price relative to the disposable income of potential consumers. Price represents a combination of the costs to produce, distribute, and market alcohol, as well as local taxes and the costs of services. Physical availability is a function of the local environment [15] and is primarily determined by licensing laws, efforts to curb illegal sales and consumption, and the alcohol outlet location, physical characteristics, and spatial access. In the spatial sciences, access generally refers to the proximity of an outlet to individuals or areas to be served [25, 26]. Thus, measures of access often utilize absolute geographic locations (latitude and longitude) or geographic base files that include small areas (e.g., block groups) or cadastral data (e.g., parcel data).

Understanding the nuances of physical availability and access is important for gaining insight into neighborhood processes related to alcohol outlets. For example, emerging research suggests a complex matrix of neighborhood ecological conditions can condition the effects of alcohol availability. For example, Pridemore and Grubesic found that greater social organization reduces the impact of alcohol outlet density on violence [27] and that local land uses like single family housing or public housing weaken and strengthen, respectively, the association between alcohol outlet density and violence ([6], see also [7–10, 28–32]).

### Measuring access

As detailed by Holmes et al. [19], there are a variety of approaches for capturing access to alcohol for a community and its residents, including outlet counts, outlets weighted by area, outlets weighted by population, outlets weighted by roadway miles, and outlets weighted by sales. While each of these is unique, all generally conform to *container* indices [33] expressed as follows for a location *i*:

1 $$ {\displaystyle {\sum}_{j\in {N}_i}{s}_j} $$

where the number of outlets, *s*_*j*_, is summed over a neighborhood *N* associated with location *i*. This can be adjusted to reflect any planning unit (e.g., tracts, block groups, etc.) and any type of outlet (e.g., on-premise, off-premise, etc.). Higher values suggest greater outlet concentration regardless of how they are standardized.

The two most popular container-based approaches for capturing alcohol outlet density include normalizing outlet counts by total population (e.g., per 1000 persons) and roadway length (e.g., 100 or 1000 miles) [34–39]. Although these measures are convenient and relatively easy to calculate, there are some theoretical and operational problems in using these approaches. The basic premise for using a per capita measurement is straightforward. The local population of an administrative unit, such as a block group, is used with outlet counts to measure residents’ exposure to outlets (e.g., three outlets per 1000 persons). The problem with this approach is two-fold. Per capita measures make an implicit assumption that outlet patrons come solely from the administrative unit in question. As detailed by Pridemore and Grubesic [6], this is not a reasonable assumption because urban residents patronize restaurants, liquor stores, and bars in areas where they do not reside. Similarly, many cities have non-residential areas (i.e., downtown business districts, areas devoted to tourism, etc.) that are full of alcohol outlets [40]. Unless one can measure the ambient population for these districts – and data are difficult to obtain and use in conjunction with traditional Census information – per capita measures can be biased. Simply put, these are locales where no residential households exist, yielding a zero-count for Census population measures and the potential for drastically biasing per capita measurements. However, they do have restaurants, bars, and other outlets that serve alcohol to the large ambient populations (e.g., tourists, employees).

The roadway miles metric is similar. For a given administrative unit, counts of alcohol outlets are used in conjunction with total roadway miles to calculate outlet density. The theory behind this is that alcohol is typically obtained through the road system (i.e., driving to alcohol outlets), so streets provide a good surrogate for population exposure to outlets. This metric is population agnostic [39]. One could double the local population of an administrative unit (or reduce it by half) and the score on the measure would not change. A key limitation is that many administrative units (e.g., ZIP codes, Census tracts, block groups) exhibit significant variability in their morphological structure and settlement patterns. Consider, for example, Census tracts in California in 2010 (*n* = 7049). Their median size is 1.17 square miles, but because street networks largely reflect settlement patterns there are no guarantees of homogeneity in the distribution of streets, especially for larger administrative units (e.g., >3 square miles). This includes over 25% of all tracts in the state. As a result, this measure of exposure is likely to have high levels of local variability and exhibit local clustering. A second limitation is that roadway density does not accurately reflect the actual process of a patron traveling from his/her residence to an alcohol outlet. Travel modes (e.g., walking, biking, driving, public transit) [41], the built environment [42], socioeconomic status [43] and other factors also influence route choice between origins and destinations.

A second popular category of approaches is proximity measures. Both Euclidean [14, 30, 31] and network distance [19, 32] proximity measures capture cost for travel between a location and outlets or cluster of outlets. Proximity is often expressed as:

2 $$ {{\displaystyle {\sum}_jd}}_{ij} $$

where *d*_*ij*_ is the distance between location *i* and outlet *j*. For example, Schonlau et al. [38] calculated the network distance between survey respondents’ home address and the nearest alcohol outlet. Variations include obtaining average values between administrative units and a subset of outlets or the use of a minimum distance measure, *min*{*d*_*ij*_}, that can be used to capture equity in service of some type of facility like a playground [33]. Unlike a container index, lower values of proximity measures mean easier access. An important limitation of these measures is that outliers can skew findings, especially if there are several large distance relationships between a location and outlets. These types of measures also fail to account for any aggregate drawing power of outlets for a region, where larger outlets (or more of them) have more attraction power (i.e., an ability to draw more patrons). Such agglomerations of outlets often exhibit more competitive pricing, a better product mix, and longer operating hours.

While container and proximity-based measures have their strengths and weaknesses, the integration of both techniques offers potential for more insight into alcohol outlet access. There are many ways this could be done. We will describe a gravity-based approach [44, 45] to measure access potential and illustrate why the disentanglement of access and availability is critical to advancing the research on alcohol control and public health. Our results will reveal massive spatial heterogeneities in alcohol access between neighborhoods, underscoring the need to avoid one-size-fits-all policies for alcohol beverage control and licensing.

## Methods

### Study area and data

We used Seattle, Washington as our study area. There are a number of reasons Seattle provides an excellent location for this study. It is a large city (~650,000 residents) and one of the fastest growing major cities in the US [46]. It has a growing economy and its diverse topography (e.g., Lake Washington, hilly, functioning harbor) and urban morphology (it is organized under a system of districts and neighborhoods [47]) creating a city of neighborhoods with many distinctive local identities [48].

We gathered data on characteristics of block groups from 2010 Census estimates provided by ESRI. These data are standardized to year 2000 Census geographic units. This choice was driven by the need to maintain data continuity with related projects. More importantly, one could easily use American Community Survey (ACS) data and 2010 block group boundaries with similar success. Block groups are the smallest administrative unit for which population and socioeconomic data are publicly available. Block groups also provide a higher resolution than larger geographic units like Census tracts or ZIP codes.

We obtained alcohol outlet data, for the entire state, from the Washington State Liquor Control Board (WSLCB) for 2010 [49]. These data included business names, license types, and street addresses, amongst many other fields. Note that Initiative I-1183 ended the state’s monopoly on liquor retailing and allowed the private sector to begin selling liquor in June 2012. However, we used 2010 data to capture a conservative benchmark of alcohol access prior to privatization in 2012 and to provide the statistical foundation for a future longitudinal study. We processed each outlet using an ESRI geocoding engine and further assessed the geographic accuracies of the latitude and longitude coordinates generated for each outlet using a hybrid semi-automated approach to ensure the minimization of spatial error and uncertainty [50]. We used for analysis only those outlets for which we were able to assign a verified, street-level match (*n* = 2402, 97.4 % of the total).

We used multiple measures of alcohol outlets. Our container-based measures for each block group included outlet counts, outlets per square mile, outlets per 10,000 residents, and outlets per 1000 feet of roadway. Our gravity potential measure [27, 39] of alcohol access blended container and proximity measures and is structured as follows:

3 $$ {Z}_i=f\left(j\epsilon {N}_i,{s}_j,{p}_i,{d}_{ij}\right) $$

where *f*( ) is a function, *N*_*i*_ is the set of outlets in a predefined neighborhood of location *i*, *s*_*j*_ is the number of outlets^1^, *p*_*i*_ is the population of area *i*, and *d*_*ij*_ is the street network distance between location *i* (block group polygon centroid) and outlet *j*. Because we are interested in proximity and a relationship where interaction decreases with distance, we used the following functional form for each location *i*:

4 $$ {Z}_i={\displaystyle {\sum}_{j\epsilon {N}_i}}\frac{s_j{p}_i}{d_{ij}^{\beta }} $$

where the friction parameter *β* reflects distance decay effects and the other parameters are as previously defined. This is a basic gravity model [33, 51–53], where interaction levels are proportional to the product of the numerator and inversely proportional to the intervening distance.

Anselin and Talen [33] indicate that there can be problems in cases where self-potential (*d*_*ij*_ = 0) is possible. We implemented a pre-processing procedure and found no outlet locations overlapped with block group centroids. Another major challenge with gravity models is determining values for *β*. This parameter dictates the steepness of the slope in a distance decay curve. Steep slopes indicate the rapid decay of potential interaction. Typically *β* is based on known or assumed travel behavior for a region [52], but this information is not always available, nor will it necessarily scale appropriately for fine-grained studies that assess access. Cross-sectional analyses can also be difficult because appropriate *β* values could vary between locations. To address this, in our analyses we assumed *β* = 1, treating distance as a linear function.

An innovation in the gravity potential measure detailed above (4) is the ability to formally evaluate different neighborhood structures (*N*_*i*_). One approach for operationalizing this is to define different distance ranges for evaluating access to outlets for different locations. This enables neighborhood spatial effects to be assessed, helping to highlight the importance of local context. For example, we set the distance limits for this analysis as 0.25, 0.50, 0.75, 1, 2, and 5 miles. All the distance limits are based on street network distance. This represents a realistic spectrum of ranges that consumers might travel to obtain alcohol, from a *micro-level* trip to the neighborhood bar or corner market (0.25 – 0.50 miles), to a *meso-level* (0.75 – 1 miles) or *macro-level* (2 – 5 miles) journey to a popular bar district or volume-based alcohol retailer.

**Fig. 1:**
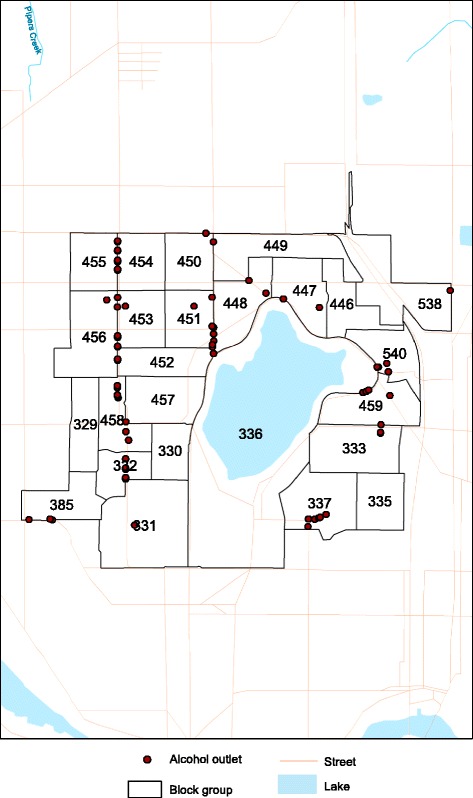
A local distribution of alcohol outlets

To illustrate the utility of the detailed gravity potential measure, a sample of alcohol outlets and block groups for a neighborhood is shown in Fig. 1. If container indices are utilized to measure alcohol access for the displayed block groups, the block group labeled 330 would be classified as having zero access even though the outlets in the block groups 332 and 458 are only 0.3 miles away. This underscores the problems with container indices. Just because a block group is devoid of outlets, it does not mean that there is no access to alcohol for its residents. Alternatively, if proximity-based approaches, such as minimum distance from block group centroids to an outlet, are used to measure alcohol access, the results are also misleading. Consider block group 446, which is completely devoid of outlets. The minimum distance from its centroid (not shown) to its nearest outlet (located in block group 447) is 0.22 miles. In this situation, block group 446 is tagged with the exact same minimum distance measure as block group 337 (0.22 miles), though block group 337 contains more outlets than any other block group in this example. This underscores the problems with simplistic proximity measures that fail to account for local concentrations of outlets. The proposed gravity potential measure overcomes these operational biases by incorporating both area concentration and physical proximity at various geographic scales (i.e., 0.25, 0.50, 0.75, 1, 2, and 5 miles). Additionally, the gravity-potential measure uses the underlying transportation network to evaluate physical access. As an example, while the Euclidean distance between block groups 452 and 459 is 1.5 miles, the actual travel distance along the street network is 3.1 miles because the lake creates a geophysical barrier. Clearly, the proposed gravity potential measure provides flexibility to evaluate a diverse range of access measures, and to reduce biases associated with commonly used container- or proximity-based approaches.

Once we calculated these access measures for each block group, we applied a local indicator of spatial association (LISA; Local Moran’s *I*) [54] to develop a functional taxonomy of potential access to alcohol for the city by block group. The LISA statistic, which is one of the most popular and easily implemented techniques for spatial cluster detection, can be used to identify spatial patterns of alcohol access, deriving a functional taxonomy of potential access to alcohol. We did this for each access metric across a range of measurement parameters and spatial weights matrices as shown in Table 1. To ensure robust results in generating our taxonomy we used several different spatial weights matrices, including queen contiguity (i.e., contiguous administrative units defined by edges or vertices touching) and a series of nearest neighbor weights matrices (i.e., *k* = 4, *…, k* = 7)^2^. The nearest neighbors are determined based on street network distance as well. Our taxonomy placed block groups into one of five categories:

**Table 1:** Access measures and associated parameters for developing a spatial typology

Measure	queen	*k = 4*	*k = 5*	*k = 6*	*k = 7*	0.25 miles	0.50 miles	0.75 miles	1 mile	2 mile	5 mile
Outlet counts	•	•	•	•	•	-	-	-	-	-	-
Outlet density (square miles)	•	•	•	•	•	-	-	-	-	-	-
Outlet density (roadway miles)	•	•	•	•	•	-	-	-	-	-	-
Outlets per capita	•	•	•	•	•	-	-	-	-	-	-
Gravity potential	•	•	•	•	•	•	•	•	•	•	•

- *High-High “access hub”*: Where block groups displaying higher levels of potential access to alcohol than the average are surrounded by other block groups with similar values. These hub areas are primarily found in core entertainment districts.
- *Low-Low “access periphery”*: Where block groups displaying lower levels of potential access to alcohol than the average are surrounded by other block groups with similar values. These areas typically correspond to large industrial zones, parks, and/or peripheral residential areas.
- *Low-High “access void”*: Where block groups displaying lower levels of potential access to alcohol than the average are surrounded by block groups displaying relatively high values. These areas are typically located near access hubs but are lacking alcohol outlets and/or significant residential populations.
- *High-Low “access archipelago”*: Where block groups displaying higher levels of potential access to alcohol than average are surrounded by block groups displaying relatively low levels. These locations typically correspond to neighborhood commercial centers that display a mix of retail and dining establishments.
- *Not Significant*: There is no statistically significant clustering of access type by block groups.

We used Cohen’s [55] Kappa coefficient, k, to evaluate the level of agreement in block group classifications for our derived taxonomies. Traditionally, k is used to measure the extent to which different raters assign the same score to the same variable. For example, two raters might be tasked with rating the condition of a house using a scale of 1) poor, 2) average, 3) good, and 4) excellent. For our purposes, we used the LISA statistic as the rater for each pairwise combination of access measures and k as the amount of classification overlap in block groups for each pairwise combination. Much like correlation statistics, the range of k is between −1 and +1. As values approach ±1, the level of agreement between raters nears perfection. As values approach 0, the amount of agreement reflects random chance. The basic interpretation of k is detailed in Table 2 [56].

**Table 2:** Interpretation of k

Value of kappa	Level of agreement
0.0-0.20	None
0.21-0.39	Minimal
0.40-0.59	Weak
0.60-0.79	Moderate
0.80-0.90	Strong
Above 0.90	Almost perfect

## Results

### Geovisualization

**Fig. 2:**
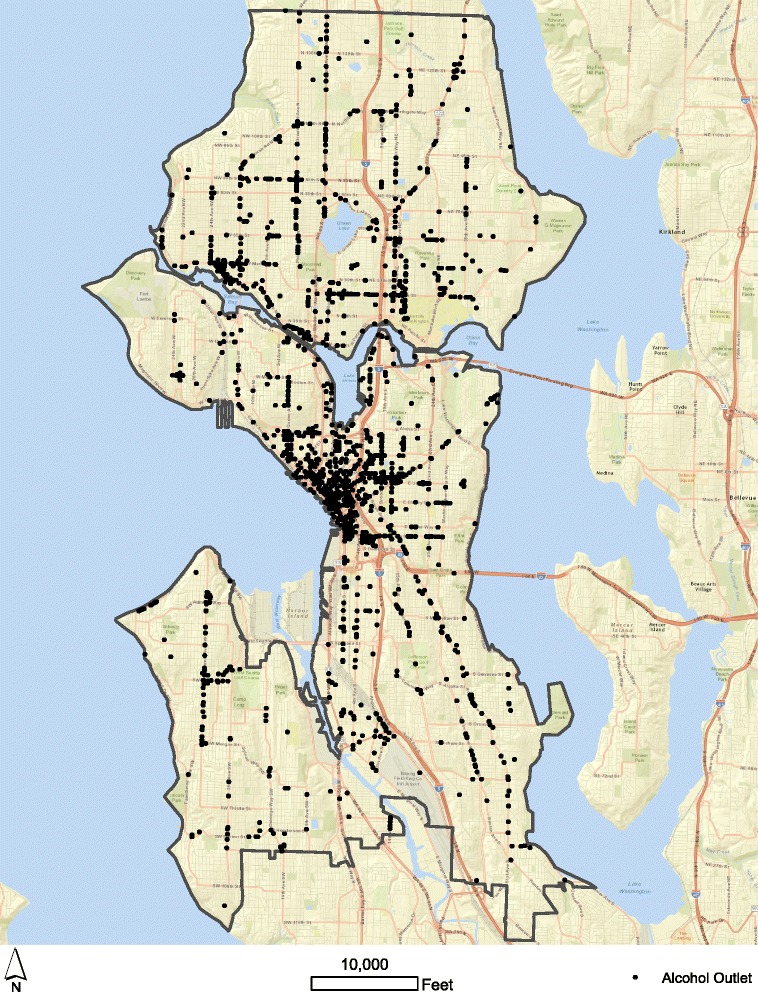
Alcohol outlets in Seattle, 2010

**Fig. 3:**
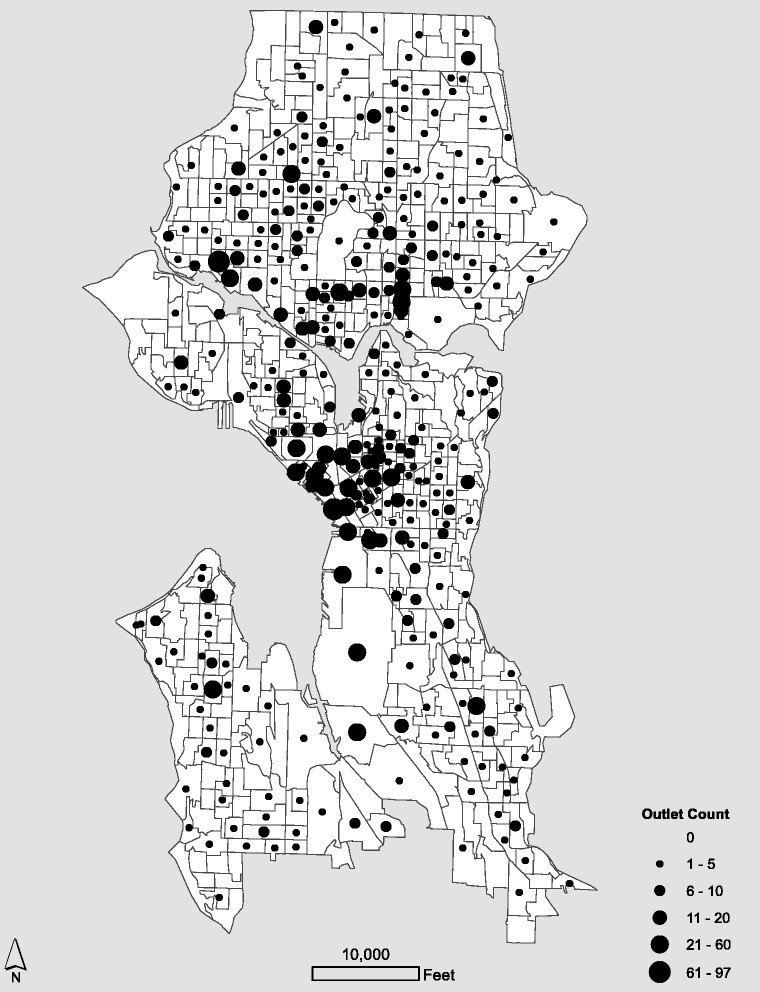
Outlet counts by block group, Seattle 2010

Figure 2 displays the spatial distribution of alcohol outlets for Seattle in 2010 (*n* = 2402) and Fig. 3 shows the aggregate spatial distribution of outlets by block group (*n* = 567). The median spatial density was 3.889 outlets per square mile, with an inter-quartile range of 16.05 (Q1 = 0; Q3 = 16.05), a maximum of 447.4, and a standard deviation of 43.31. The median spatial density of alcohol outlets calculated with roadway miles was 2.04 outlets per 1000 feet of roadway, with an inter-quartile range of 1.944 (Q1 = 0; Q3 = 1.944), a maximum of 48.3, and a standard deviation of 4.84. While only the alcohol outlets in Seattle are shown in Fig. 1, in an effort to mitigate edge effects, the state-wide database of alcohol outlets is employed to compute gravity potential measures.

**Fig. 4:**
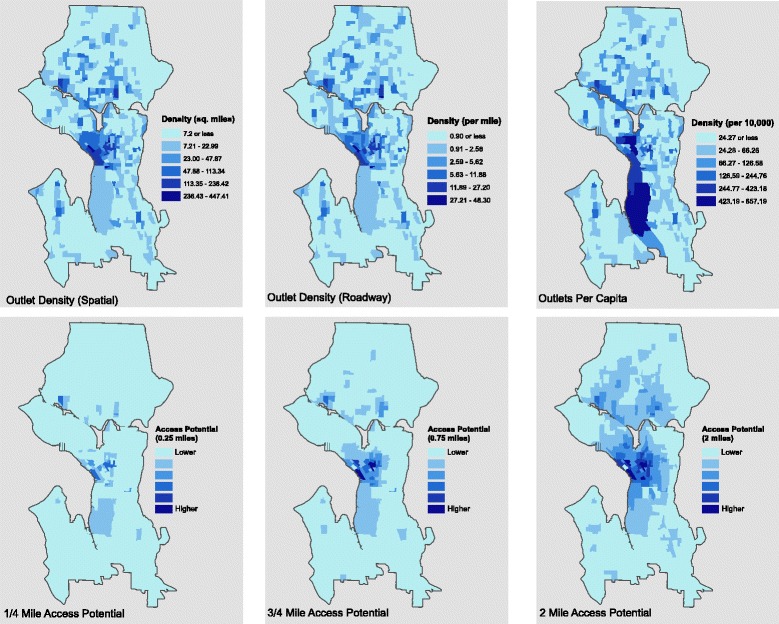
A comparison of alcohol outlet access measures, Seattle 2010

For reference, Fig. 4 displays the spatial and roadway density measures along with outlets per 10,000 residents and three gravity potential measures (0.25, 0.75, and 2 miles). The number of outlets within the predefined neighborhoods are used to compute gravity potential measures. One of the most conspicuous patterns displayed in Fig. 4 is the high per capita value for alcohol outlets associated with the large industrial district south of the Seattle central business district (CBD). These block groups possess relatively low residential populations relative to other areas of the city and thus the per capita values skew high in these areas. In previous work, some scholars removed these types of low population units for statistical analysis [57], but in an effort to retain spatial continuity we retain them. Also of note in Fig. 4 is the progressive expansion of access potential as one increases the neighborhood distance limits (e.g., from 0.25 to 0.75 and 2 miles). There are clearly large differences between a micro measure like 0.25 miles and a macro measure like 2 miles. We detail below the specific differences in these measures and their importance.

### A functional taxonomy of access to alcohol

**Fig. 5:**
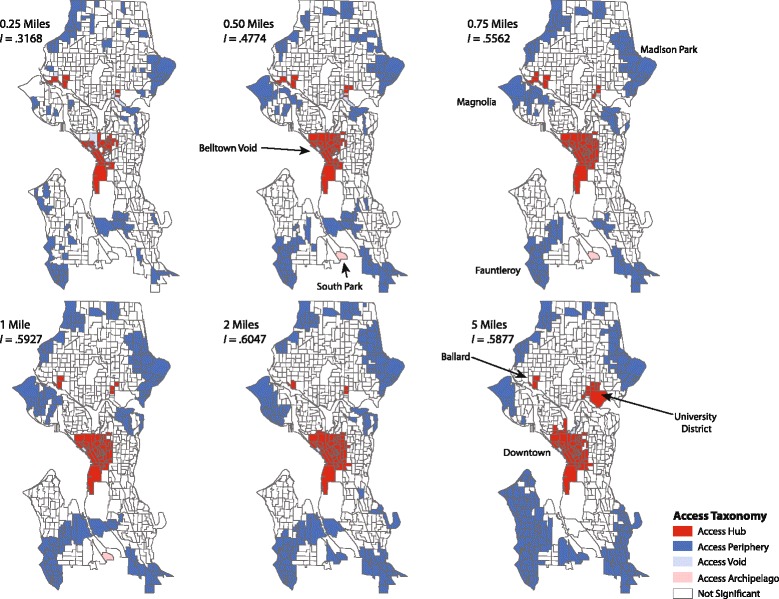
Alcohol access taxonomy for Seattle (*k* = 4), 2010

We applied a local indicator of spatial association to develop a functional taxonomy of potential access to alcohol for the city by block group. We did this for each access metric across a range of measurement parameters and spatial weights matrices. Examples are illustrated in Fig. 5, which displays the results for *micro*, *meso*, and *macro* access neighborhoods using a *k* = 4 nearest neighbor weight matrix. The choice of *k* = 4 is driven by a need for analytical clarity. Specifically, *k* = 4 ensures that the autocorrelation statistics and resulting typologies remain relatively local. As one increases the number of neighbors (e.g., *k* = 7), additional geographic smoothing of the variable of interest occurs. Depending on the morphology of a city and the topology of the underlying administrative units, this may or may not be appropriate. All six panels display moderate positive spatial autocorrelation, with the lowest (*I* = .3168) for the most compact neighborhood definition (0.25 miles) and the highest (*I* = .6047) for the 2-mile neighborhood. To accompany Fig. 5, Table 3 details the descriptive statistics (e.g., population, access potential score, etc.) of each cluster in the access taxonomy for the *k* = 4 spatial weight matrix. For example, there are 30 HH (high-high) block groups in the micro (0.25 miles) typology that as a group contain an average of 19.93 outlets, a local average population of 1,530.97, and an average spatial density of alcohol outlets of 105.95 per square mile. Intuitively, higher values for the access potential measure suggest greater levels of access and the HH group displays the largest value (326,354.59) for the 0.25 mile neighborhood. In instances where values are zero, this means that no block groups were assigned to that particular category in the local statistical tests.

**Table 3:** Descriptive statistics for the *k* = 4 alcohol access taxonomy

	Block group count	Average population	Average outlets	Average spatial density	Average access potential
Micro (0.25)					
HH	30	1530.97	19.93	105.95	326354.59
LL	67	994.49	1.87	4.21	4639.93
LH	6	1501.00	11.00	35.33	14228.53
HL	0	0.00	0.00	0.00	0.00
Micro (0.50)					
HH	41	1523.83	19.33	103.66	590171.25
LL	85	1017.34	1.59	3.00	7631.71
LH	4	1147.00	5.50	39.09	28798.49
HL	1	1906.00	9.00	15.47	121727.07
Meso (0.75)					
HH	49	1438.67	18.35	99.02	785562.90
LL	99	1016.60	1.29	2.95	14297.81
LH	1	1182.00	18.00	138.14	13052.67
HL	1	1906.00	9.00	15.47	130927.10
Meso (1.00)					
HH	46	1463.45	18.91	102.56	1041673.18
LL	107	1019.51	1.63	3.20	21851.45
LH	1	1182.00	18.00	138.14	17348.65
HL	1	1906.00	9.00	15.47	139154.97
Macro (2 miles)					
HH	49	1426.00	18.51	101.14	1542235.60
LL	113	1033.54	1.63	3.62	56247.19
LH	1	1182.00	18.00	138.14	23808.88
HL	0	0.00	0.00	0.00	0.00
Macro (5 miles)					
HH	57	1502.67	16.02	88.50	1800325.46
LL	139	1062.65	1.53	4.49	175853.57
LH	2	899.00	16.50	85.58	478306.52
HL	0	0.00	0.00	0.00	0.00

Note that the location of block groups classified as access hubs remain fairly persistent regardless of the neighborhood distance structures used. There is some variation in the spatial structure of these access hubs across the *micro*, *meso*, and *macro* spectrum, but downtown Seattle, the University District, and the Ballard neighborhood (which is one of the more popular dining and entertainment districts in the city), are consistently areas with high levels of potential access to alcohol outlets for Seattle. Figure 5 also illustrates that the spatial structure of the access periphery is more mutable, but as one would expect there is a strong correlation between these locales and large green spaces and industrial districts where residential population and establishments are sparse. Also of interest is the emergence of an access archipelago for the 0.50, 0.75, and 1-mile neighborhoods (see the 0.50 panel for details). This corresponds to a portion of the South Park neighborhood of Seattle, which is home to a satellite Boeing Company campus, the center of Seattle’s Latino population, and the location of numerous alcohol outlets (primarily restaurants but also Cadence Winery and Odin Brewing). It is also worth noting that the access archipelago for 0.50-mile neighborhood becomes access periphery for the 2- and 5-mile neighborhood. This is driven by a smoothing process that occurs when distance limits for a neighborhood increases. In short, a block group that displays relatively high levels of potential access for a very local neighborhood (e.g., 0.50 miles) may not be particular high relative to other block groups that are included in larger neighborhoods. Finally, there is a persistent access void in downtown Seattle that corresponds to the block group associated with the northern terminus of the Alaskan Way Viaduct, a double-decked portion of State Route 99 that runs along Seattle’s western industrial district and waterfront. Although the viaduct empties into Belltown, Seattle’s most densely populated neighborhood and largest entertainment district, the area just south of where Route 99 meets Battery Street has far fewer alcohol outlets than its neighboring block groups. In part, this is explained by the presence of StamLab (University of Washington’s Genome Science research center), Storbeck Park, and several other corporate campuses that significantly reduce the available commercial space for alcohol outlets. The area is not devoid of alcohol outlets, but when compared to the remainder of Belltown, its access potential is much lower^3^.

Although space limitations prevent us from detailing and visualizing the results associated with alternative spatial weights matrices for the access potential measure, or different values for *β*, the core patterns shown in them are the same. Downtown (including Belltown), the University District, and Ballard remain access hubs, neighborhoods like Madison Park (home to the Washington Park Arboretum) and Magnolia (home to Discovery Park) remain part of the access periphery, as do portions of southwest Seattle (including the Fauntleroy neighborhood).

### Inter-rater agreement

Given the relative consistency of the gravity potential measure across neighborhood structures and spatial weight matrices, an important next step is to provide an unbiased statistical comparison of this gravity potential measure to the more popular and widely-used metrics for capturing access to alcohol outlets. Table 4 shows the results of this analysis via Cohen’s Kappa coefficient, k. To ease interpretation, cells in Table 4 are shaded with a choropleth scheme, with darker shades representing higher levels of agreement (corresponding to Table 2).

**Table 4:**
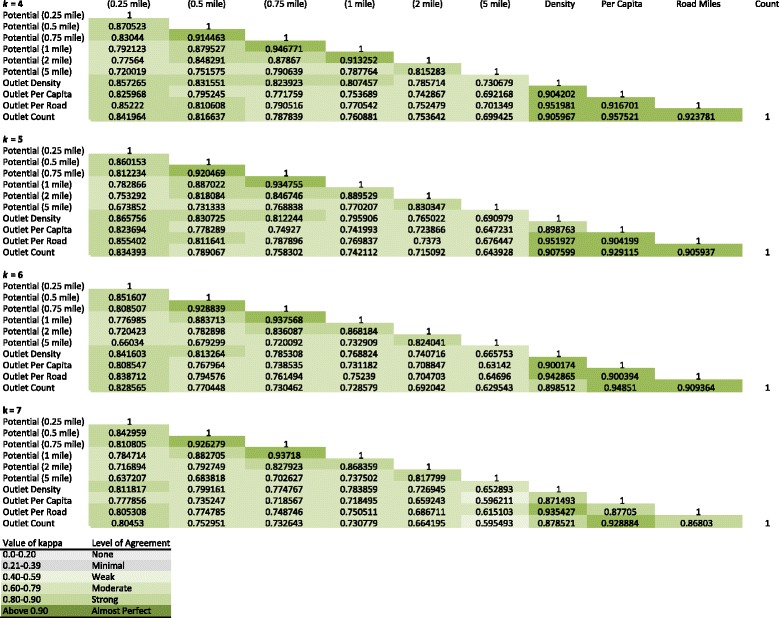
Cohen’s Kappa coefficient, k

There are several interesting patterns worth noting. First, as one might expect, access potential values that are similar in spatial structure share relatively high k values. For example, when *k* = 4, the k value for 0.75-mile and 1-mile neighborhoods is 0.95, suggesting a high level of agreement between the two LISA classifications and their associated pattern. A similarly strong level of agreement is found in the 1- and 2-mile neighborhoods (0.91). This is a pattern that is perpetuated throughout the various spatial weight matrices (*k* = 4, …, 7). Second, the more traditional outlet density measures (spatial, per capita, roadway, and raw counts) also share moderate to strong high k values amongst themselves. Again, when *k* = 4, the spatial density and roadway density measures have a k value of 0.95 (roadway). Similarly, the per capita and raw count measures have a *k* value of 0.96. This is not unexpected given the nature of these container measures.

**Fig. 6:**
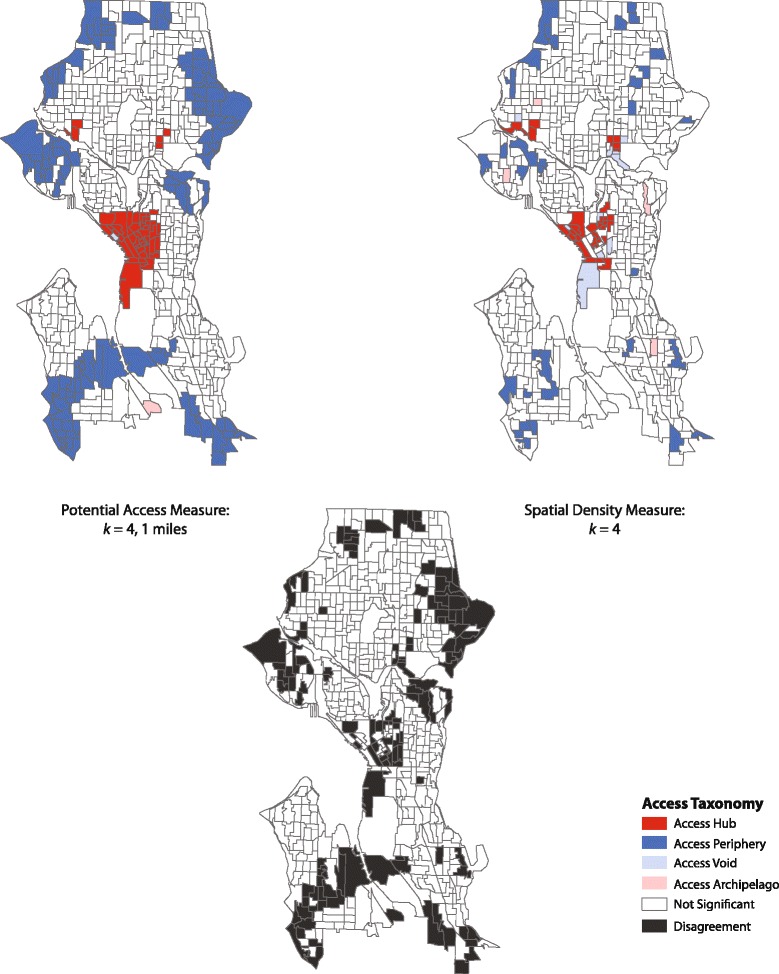
Overlap in spatial taxonomy between a 1 mile potential access measure and a standard spatial density measure

The most important feature of Table 4 is that the k values for our gravity potential measure and the traditional measures display a marked divergence. For example, consider the 1-mile access gravity potential measure when *k* = 4. There is strong agreement between similarly structured access measures (e.g., 0.95 with the 0.75-mile measure). However, this level of agreement declines when comparing the 1-mile access measure with raw counts (0.76) and with outlets per square mile (0.81), per 10,000 residents (0.75), and per 1000 feet of roadway (0.77). This suggests there is some overlap in the derived taxonomies but that the overall patterns are not in complete agreement. Figure 6 highlights these differences using the 1-mile gravity potential measure and the spatial density measure. There are clear differences in the downtown area and the more peripheral areas of the city, especially near the Madison Park, Magnolia, and Fauntleroy neighborhoods.

## Discussion

Measuring access to alcohol outlets is a complex task, and given prior research and our results it is clear there are several facets worth further discussion. We focus our discussion on elements of measurement bias and our case for using the gravity potential measure, our spatial taxonomy of alcohol access, and our findings as they relate specifically to study area of Seattle.

### Measurement bias

Table 4 shows substantial differences between the gravity potential measures we introduce here and the traditional widely applied container-based measures. Although we expected these measures to diverge somewhat, it is surprising to see such a high level of incongruence between metrics attempting to capture the same thing – access to alcohol.

Why do these large differences exist? From an operational perspective, container-based approaches have known weaknesses. The per capita measurement for any given administrative unit assumes its alcohol outlets receive local patronage only. This is not reasonable because urban residents frequent restaurants, liquor stores and bars in areas where they do not reside, and most importantly there is variation among units in the level of patrons from outside the unit. In addition, most cities have non-residential areas (e.g., industrial districts, areas devoted to tourism) that may or may not have alcohol outlets [40]. These urban morphological traits are common to most large metropolitan areas and the use of a per capita metric to capture alcohol access can lead to measurement bias. Figure 4 highlights these biases clearly for Seattle. The large dark blue block group south of downtown is low in residential population but contains a few alcohol outlets. The problem is that the per capita measurement makes these areas appear to be hot spots of alcohol access when in fact they are not. This is not only important in itself but has negative ramifications for models explaining the causes and consequences of the spatial distribution of alcohol availability.

The container-based measures for roadway miles and spatial density measures are less biased but have their own weaknesses. The spatial density measure relies on local administrative units (e.g., block groups, ZIP codes) for density calculations. This is not inherently a problem, but ZIP codes are highly dynamic [58, 59] and can create spatial mismatch problems over time. Similarly, although the US Census Bureau tries to standardize block group population size, their spatial size is more variable throughout a metropolitan area. This means that block groups that are spatially large and contain many alcohol outlets may not appear to be as important as block groups that are spatially smaller and contain fewer outlets. Not only are such results misleading, the lack of contextual awareness for the spatial density measure begs for improvement. The roadway miles measure is similarly limited. It is sensitive to variability in local settlement patterns and to the morphological structure of administrative units. More importantly, roadway density does not capture or accurately measure the actual process of a patron traveling from his/her residence to an alcohol outlet. Finally, both measures are population agnostic, but why would one want to ignore the most important aspect of alcohol access: the consumer? Although the spatial and roadway density measures remove the local patronage bias detailed above, they create poor contextual frameworks for capturing access because they largely ignore the underlying spatial processes and dynamics of consumer demand for a region.

Given the known problems associated with container indices, and those with basic proximity measures detailed earlier, we introduce the gravity potential measure to gauge access to alcohol. This measure blends the best of both techniques and provides a mechanism to capture local context and the effects of spatial interaction for estimating the physical availability of alcohol in a region. One major advantage of this gravity potential measure is its *scale specificity*. The ability to integrate multiple definitions of a neighborhood across *micro*, *meso*, and *macro* geographic scales is a powerful tool. In short, rather than using a blunt one-size-fits-all measurement framework, the inherent flexibility of the gravity potential measure can provide decision-makers with a spectrum of practical empirical information from the highly local (micro) to the more regional (macro). A second major advantage is that the gravity potential measure uses the underlying transportation network to evaluate access. Rather than relying upon some type of Euclidean distance metric (e.g., distance buffers around outlets), which can ignore the important local context of urban environments (e.g., geophysical barriers such as waterways and green spaces), the gravity potential measure leverages information on how consumers actually travel within the urban environment along street networks. This is also true of some basic proximity measures, but because the gravity potential measure includes a *β* parameter that accounts for distance decay effects, it can be adjusted to best reflect known or assumed travel behavior for a region.

### Access taxonomy

A second major feature of our results is our development of the alcohol access taxonomy. We derived our taxonomy by combining the results for our gravity potential measure with local indicators of spatial association. The resulting taxonomy included *access hubs*, *access voids, access archipelagos*, and the *access periphery*. Access hubs correspond to hot spots of alcohol outlets in a region that are dense with bars, restaurants, and other entertainment venues where alcohol may be served (e.g., sports stadiums). Access voids correspond to areas where some outlets may be present but the overall level of access within the void is much lower compared to surrounding areas. This may be a function of land use, a small residential population, or some other combination of contextual factors. Access archipelagos correspond to regions where access to outlets is much higher than surrounding areas. Many factors may drive this including clusters of restaurants or local retailers serving an isolated residential and/or daytime business population. Finally, the access periphery is composed of areas that on average have far fewer alcohol outlets than their neighbors. This periphery can take many forms, including regions dominated by parks, greenspace, or industry. In many cases, the access periphery simply corresponds to low density residential areas where there is no mixing of commercial land use.

There are three major advantages in using this taxonomy for evaluating alcohol access. First, the ability to statistically differentiate between these zone types and to highlight the peaks and valleys of alcohol access within a region can help decision-makers understand how the licensing process for outlets has manifested over time. Second, this type of geospatial intelligence can facilitate a more efficient and equitable licensing process. Many cities have limits on the density and/or location of alcohol outlets [60]. The ability to monitor changes in access hubs or access archipelagos provides communities and alcohol licensing boards with the surveillance tools necessary to ensure sensitive areas are not overloaded with outlets. This is especially true for vulnerable neighborhoods where there is an association between alcohol outlets and violence [27] or levels of alcohol-related morbidity and mortality are high. Such monitoring efforts empower communities to deny license renewals for known problematic outlets using defensible empirical evidence rather than subjective evaluations. For example, where Seattle is concerned, there is an instance where the spatial density of outlets is 447 per square mile. Is this too much? Perhaps, but this is a decision that the residents, policymakers, and law enforcement agencies within Seattle need to make. Third, this framework is easily portable between areas. Thus, the ability to compare the spatial structure of access hubs within and between regions can provide a powerful benchmarking tool for understanding spatial variation in access to alcohol, the causes and consequences of this variation, and alcohol policy.

### Alcohol access in Seattle

Our results for Seattle revealed three major access hubs: downtown, the University District, and Ballard. This does not mean these neighborhoods have the highest aggregate counts of outlets, and Fig. 3 clarifies this. Instead, their status as hubs represents a combined measure of outlet presence, proximal residential population, and travel distance. Our results were robust across a range of spatial weight matrices and neighborhood definitions. While these results were not surprising given the Seattle dining and entertainment scene, the presence of a relative access void near Belltown and the emergence of an access archipelago in South Park were unexpected. The relatively large presence of an access periphery within the city is noteworthy. The east and west sides of Seattle are surrounded by water. This physical boundary drastically limits the physical availability of alcohol to residents of neighborhoods like Madison Park, Magnolia, Fauntleroy, and others along the city’s waterfronts of Lake Washington, Puget Sound, and Lake Union, which reiterates the importance of geospatial context in evaluating alcohol access for a region. The use of standard density measures or Euclidean distance metrics simply cannot detect these geophysical nuances.

### Limitations and implications

There are several limitations to this study that require acknowledgment. First, with the recent privatization of liquor sales in the state of Washington, the landscape of alcohol access has changed. We purposely used pre-privatization data from 2010 to illustrate this method and to provide a benchmark for tracking the changes brought about by privatization that we are pursuing in other work. Second, it is important to acknowledge that several parameters associated with the access potential measure can be changed to reflect alternative representations of outlet attractiveness based on size, product variety, or distance decay (*β* = 1, 1.5, 2). For our analysis of Seattle we conducted basic sensitivity analyses on the beta parameter. Table 5 shows the sensitivity results via Cohen’s Kappa coefficient for different values of beta. Specifically, the values of 1.0, 1.5, and 2.0 are used to compute the gravity potential measures across all neighborhood definitions of 0.25, 0.75, 1, 2, and 5 miles. The block groups are then classified based on the LISA statistics of gravity potential measures and the Kappa coefficients are computed to evaluate the level of agreement in block group classifications for different betas. As Table 5 shows, there were moderate to strong levels of agreement for betas 1 and 1.5, and betas 1.5 and 2.0, while weak to moderate agreement can be observed for betas 1 and 2. Also, as the geographical scale of neighborhood increases, the level of agreement generally decreases, indicating that the impacts of beta are relatively small for the micro neighborhood but large for the meso and macro neighborhood. That said, while there were modest variations in block group classifications, the core patterns remained consistent with what were reported in previous section. This may not be true for regions that have different local morphologies and travel behaviors. We also explored the use of alternative spatial units for analysis, including Census blocks. Again, although there were slight variations, the patterns for both blocks and block groups remained consistent. Lastly, it is worth noting that the lack of local behavioral information, especially when it comes to trip generation, make it difficult to apply a uniform distance limit to neighborhood definitions. Readers may wish to explore the work of Zhang et al. [61, 62] for examples of best practice in this domain.

**Table 5:** Sensitivity analysis of beta values

	Potential (0.25 mile)	Potential (0.5 mile)	Potential (0.75 mile)	Potential (1 mile)	Potential (2 mile)	Potential (5 mile)
beta 1 & 1.5	0.892042	0.814498	0.773682	0.80866	0.852235	0.732094
beta 1 & 2.0	0.728637	0.714258	0.642138	0.615308	0.607277	0.457819
beta 1.5 & 2.0	0.799089	0.861384	0.848512	0.78557	0.758577	0.676528

## Conclusion

The public health, law enforcement, and policy implications of alcohol sales, access, and consumption create a range of challenges for communities. Concentrations of outlets appear to be associated with antisocial behavior, violence, sexually transmitted diseases, reduced productivity at work, alcohol-related injuries, and neighborhood quality of life. There are limitations to measuring spatial access to alcohol, however, and so our purpose was to introduce and demonstrate measure of alcohol access that improves upon the limitations of prior metrics and to provide an empirically derived taxonomy of areas based on their type of alcohol access. The gravity potential measure provides a more realistic and empirically defensible tool for evaluating alcohol access and it provides more flexibility and usable information for decision-makers through the ability to incorporate scale-specific neighborhood definitions and alternative spatial weights matrices for generating spatial taxonomies of access. The gravity potential measure and the empirically derived taxonomy should not only benefit public health, geography, criminology, and public policy communities as it relates to alcohol, but these tools are easily portable and can be used to examine similar topics as they relate the presence of nuisance facilities like payday lenders, pawn shops, public transportation nodes, abandoned structures, and certain types of land use.

## Footnotes

1 Although we are using the total number of outlets for *s*_*j*_ in this application, outlet size (e.g., total square footage) could also be used if that information was available.

2 Alternatively, the choice of *k*-nearest neighbor values could be informed by exploring the median and average number of neighbors for the block groups within a region.

3 It is important to highlight that the transportation network in this part of the city, especially the Alaskan Way Viaduct and all of the nearby one-way streets, are likely complicating network computations. Thus, readers should view this as a conservative estimate of access potential for the Belltown block group. For patrons walking on foot or bicycling, this section of Belltown may exhibit higher levels of local access potential.

## References

[1] Block RL, Block CR (1995). Space, place and crime: hot spot areas and hot places of liquor related crime. Crime Prevention Studies.

[2] Roman CG, Reid SE, Bhati AS, Tereschenko B. Alcohol outlets as attractors of violence and disorder. The Urban Institute. 2008. http://www.urban.org/publications/411663.html. Accessed 27 Apr 2015.

[3] Britt HR, Carlin BP, Toomey TL, Wagenaar AC (2005). Neighborhood level spatial analysis of the relationship between alcohol outlet density and criminal violence. Environ Ecol Stat.

[4] Speer PW, Gorman DM, Labouvie EW, Ontkush MJ (1998). Violent crime and alcohol availability: relationships in an urban community. J Public Health Pol.

[5] Zhu L, Gorman DM, Horel S (2004). Alcohol outlet density and violence: a geospatial analysis. Alcohol Alcohol.

[6] Pridemore WA, Grubesic TH (2012). A spatial analysis of the moderating effects of land use on the association between alcohol outlet density and violence in urban areas. Drug Alcohol Rev.

[7] Grubesic TH, Pridemore WA, Williams DA, Philip-Tabb L (2013). Alcohol outlet density and violence: the role of risky retailers and alcohol-related expenditures. Alcohol Alcohol.

[8] Lugo W (2008). Alcohol and crime: beyond density. Secur J.

[9] de Vocht F, Heron J, Angus C, Brennan A, Mooney J, Lock K, Hickman M. Measurable effects of local alcohol licensing policies on population health in England. J Epidemiol Community Health. 2016;70(3):231-37. 10.1136/jech-2015-206040 PMC4789824 26555369

[10] Livingston M (2008). A longitudinal analysis of alcohol outlet density and assault. Alcohol Clin Exp Res.

[11] Chesson H, Harrison P, Kassler WJ (2000). Sex under the influence: the effect of alcohol policy on sexually transmitted disease rates in the United States. J Law Econ.

[12] Jones S, Casswell S, Zhang JF (1995). The economic costs of alcohol‐related absenteeism and reduced productivity among the working population of New Zealand. Addiction.

[13] West R, Drummond C, Eames K (1995). Alcohol consumption, problem drinking and antisocial behaviour in a sample of college students. Brit J Addict.

[14] Wilkinson C, Livingston M (2012). Distances to on- and off-premise alcohol outlets and experiences of alcohol-related amenity problems. Drug Alcohol Rev.

[15] Stockwell T, Gruenewald PJ. Controls on the physical availability of alcohol. In: The essential handbook of treatment and prevention of alcohol problems. Editors by Nick Heather and Tim Stockwell. New York: John Wiley and Sons; 2004. p. 213–233.

[16] Livingston M, Chikritzhs T, Room R. Changing the density of alcohol outlets to reduce alcoholrelated problems. Drug Alcohol Rev. 2007;26:557–66. 10.1080/09595230701499191 17701520

[17] Snowden AJ, Pridemore WA (2013). Alcohol and violence in a nonmetropolitan college town alcohol outlet density, outlet type, and assault. J Drug Issues.

[18] Angelucci M. Love on the rocks: Domestic violence and alcohol abuse in rural Mexico. BE J of Econ Anal Poli. 2008;8(1).

[19] Holmes J, Guo Y, Maheswaran R, Nicholls J, Meier PS, Brennan A (2014). The impact of spatial and temporal availability of alcohol on its consumption and related harms: a critical review in the context of UK licensing policies. Drug Alcohol Rev.

[20] Graves SM (2003). Landscapes of predation, landscapes of neglect: a location analysis of payday lenders and banks. Prof Geogr.

[21] Kubrin CE, Squires GD, Graves SM, Ousey GC (2011). Does fringe banking exacerbate neighborhood crime rates?. Criminol Public Policy.

[22] Loukaitou-Sideris A (1999). Hot spots of bus stop crime: the importance of environmental attributes. J Am Plan Assoc.

[23] Spelman W (1993). Abandoned buildings: magnets for crime?. J Crim Just.

[24] Stucky TD, Ottensmann JR (2009). Land use and violent crime. Criminology.

[25] Murray AT, Davis R (2001). Equity in regional service provision. J Regional Sci.

[26] Matisziw TC, Grubesic TH (2010). Evaluating locational accessibility to the US air transportation system. Transport Res A-Pol.

[27] Pridemore WA, Grubesic TH (2012). Community organization moderates the effect of alcohol outlet density on violence. Br J Sociol.

[28] Humphreys DK, Eisner MP (2014). Do flexible alcohol trading hours reduce violence? A theory-based natural experiment in alcohol policy. Soc Sci Med.

[29] Day P, Breetzke G, Kingham S, Campbell M (2012). Close proximity to alcohol outlets is associated with increased serious violent crime in New Zealand. Aust N Z J Public Health.

[30] Pollack CE, Cubbin C, Ahn D, Winkleby M (2005). Neighbourhood deprivation and alcohol consumption: does the availability of alcohol play a role?. Int J Epidemiol.

[31] Theall KP, Scribner R, Cohen D (2009). The neighborhood alcohol environment and alcohol-related morbidity. Alcohol Alcohol.

[32] Kavanagh AM, Kelly MT, Krnjacki L (2011). Access to alcohol outlets and harmful alcohol consumption: a multi-level study in Melbourne, Australia. Addiction.

[33] Talen E, Anselin L (1998). Assessing spatial equity: an evaluation of measures of accessibility to public playgrounds. Environ Plann A.

[34] Cohen DA, Ghosh-Dastidar B, Scribner R, Miu A, Scott M, Robinson P, Brown-Taylor D (2006). Alcohol outlets, gonorrhea, and the Los Angeles civil unrest: a longitudinal analysis. Soc Sci Med.

[35] Gruenewald PJ, Freisthler B, Remer L, LaScala EA, Treno A (2006). Ecological models of alcohol outlets and violent assaults: crime potentials and geospatial analysis. Addiction.

[36] Chen MJ, Grube JW, Gruenewald PJ (2010). Community alcohol outlet density and underage drinking. Addiction.

[37] Treno AJ, Gruenewald PJ, Wood DS, Ponicki WR (2006). The price of alcohol: a consideration of contextual factors. Alcohol Clin Exp Res.

[38] Schonlau M, Scribner R, Farley TA, Theall KP, Bluthenthal RN, Scott M, Cohen DA (2008). Alcohol outlet density and alcohol consumption in Los Angeles county and southern Louisiana. Geospat Health.

[39] Romley JA, Cohen D, Ringel J, Sturm R (2007). Alcohol and environmental justice: the density of liquor stores and bars in urban neighborhoods in the United States. J Stud Alcohol Drugs.

[40] LaVeist TA, Wallace JM (2000). Health risk and inequitable distribution of liquor stores in African American neighborhood. Soc Sci Med.

[41] Cervero R (2002). Built environments and mode choice: toward a normative framework. Transport Res Part D-Tr E.

[42] Handy SL, Boarnet MG, Ewing R, Killingsworth RE (2002). How the built environment affects physical activity: views from urban planning. Am J Prev Med.

[43] Pucher J, Renne JL (2003). Socioeconomics of urban travel: evidence from the 2001 NHTS. Transport Q.

[44] Knox PL (1978). The intraurban ecology of primary medical care: patterns of accessibility and their policy implications. Environ Plann A.

[45] Harris C (1954). The market as a factor in the localization of industry in the United States. Ann Assoc Am Geogr.

[46] Balk G. Census: Seattle is the fastest-growing big city in the U.S. Seattle Times. 2014. http://tinyurl.com/qzth9kb. Accessed 27 Apr 2015.

[47] Seattle City Clerk. Neighborhood Map Atlas. 2015. http://clerk.ci.seattle.wa.us/~public/nmaps/neiglist.htm. Accessed 27 Apr 2015.

[48] Findwell. Seattle Neighborhood Guide. http://seattle.findwell.com/seattle-neighborhoods/. Accessed 27 Apr 2015.

[49] Washington State Liquor Control Board. http://www.liq.wa.gov/. Accessed 27 Apr 2015.

[50] Murray AT, Grubesic TH, Wei R, Mack EA (2011). A hybrid geocoding methodology for spatio‐temporal data. Trans GIS.

[51] Fotheringham AS (1983). A new set of spatial-interaction models: the theory of competing destinations. Environ Plann A.

[52] Haynes KE, Fotheringham AS (1984). Gravity and spatial interaction models (Vol. 2).

[53] Besag J. Spatial interaction and the statistical analysis of lattice systems. J Roy Statist Soc Ser B. 1974;36(2):192-236.

[54] Anselin L (1995). Local indicators of spatial association—LISA. Geogr Anal.

[55] Cohen J (1960). A coefficient of agreement for nominal scales. Educ Psychol Meas.

[56] McHugh ML (2012). Interrater reliability: the kappa statistic. Biochem Med.

[57] Jennings JM, Milam AJ, Greiner A, Furr-Holden CDM, Curriero FC, Thornton RJ (2014). Neighborhood alcohol outlets and the association with violent crime in one Mid-Atlantic city: The implications for zoning policy. J Urban Health.

[58] Grubesic TH, Matisziw TC (2006). On the use of ZIP codes and ZIP code tabulation areas (ZCTAs) for the spatial analysis of epidemiological data. Int J Health Geogr.

[59] Grubesic TH (2008). Zip codes and spatial analysis: Problems and prospects. Socio Econ Plan Sci.

[60] Grubesic TH, Murray AT, Pridemore WA, Tabb LP, Liu Y, Wei R (2012). Alcohol beverage control, privatization and the geographic distribution of alcohol outlets. BMC Public Health.

[61] Zhang X, Hatcher B, Clarkson L, Holt JB, Bagchi S, Kanny D, Brewer R (2015). Impact of changes in on-premise alcohol outlet density on violent crime in Atlanta, Georgia, 1997–2007. Prev Chronic Dis.

[62] Zhang X, Lu H, Holt JB (2011). Modeling spatial accessibility to parks: a national study. Int J Health Geogr.

